# Hypoxia-Driven Immune Escape in the Tumor Microenvironment

**DOI:** 10.3390/cells9040992

**Published:** 2020-04-16

**Authors:** Alyssa Vito, Nader El-Sayes, Karen Mossman

**Affiliations:** 1 Department of Biochemistry and Biomedical Sciences, McMaster Immunology Research Centre, McMaster University, Hamilton, ON L8S 4K1, Canada; vitoar@mcmaster.ca (A.V.); elsayesn@mcmaster.ca (N.E.-S.); 2 Department of Pathology and Molecular Medicine, McMaster Immunology Research Centre, McMaster University, Hamilton, ON L8S 4K1, Canada

**Keywords:** hypoxia, tumor microenvironment, immunogenic cell death, therapeutics

## Abstract

The tumor microenvironment is a complex ecosystem comprised of many different cell types, abnormal vasculature and immunosuppressive cytokines. The irregular growth kinetics with which tumors grow leads to increased oxygen consumption and, in turn, hypoxic conditions. Hypoxia has been associated with poor clinical outcome, increased tumor heterogeneity, emergence of resistant clones and evasion of immune detection. Additionally, hypoxia-driven cell death pathways have traditionally been thought of as tolerogenic processes. However, as researchers working in the field of immunotherapy continue to investigate and unveil new types of immunogenic cell death (ICD), it has become clear that, in some instances, hypoxia may actually induce ICD within a tumor. In this review, we will discuss hypoxia-driven immune escape that drives poor prognostic outcomes, the ability of hypoxia to induce ICD and potential therapeutic targets amongst hypoxia pathways.

## 1. Introduction

Cancer is characterized by uncontrolled cell growth and rapid proliferation. The enhanced cellular kinetics with which cancer cells divide and grow inevitably causes nutrient depletion, as well as an influx of transcription factors and proteins responsible for the induction of hypoxia [1]. The increased proliferative capacity of malignant cells requires constant uptake of oxygen, which is a limiting factor in an oxygen-deprived environment. In response to low oxygen levels in tumors, transcriptional responses upregulate hypoxia-inducible factors (HIFs), transcriptional factors that control the expression of many angiogenic, metabolic and cell cycle genes [2]. While malignant cells are able to continue to grow and even thrive in the resultant hypoxic microenvironment, it creates inhospitable conditions for immune cells and dampens the response of key regulatory pathways, resulting in immunosuppression [3]. Interestingly, while it is well established in the literature that hypoxia contributes to a diminishing immune response [4,5,6], it has also been shown to simultaneously play an immunostimulatory role, as the consequential pro-inflammatory environment lends to cells dying in an immunogenic manner [7]. This paper discusses the complex and opposing roles of hypoxia signaling in driving immune escape, promoting tumor growth and metastatic potential, while also enhancing certain immunogenic features of the tumor microenvironment (TME).

The ability of the immune system to recognize malignant cells as foreign entities and clear them from the body has been the springboard for a wave of innovative immunotherapies that have revolutionized the way we think about treating cancer patients. As we focus on the development of new and improved therapies that can harness the potential of the immune system to detect and kill tumors, we also begin to better understand the multi-faceted biology and interactions associated with the TME. The TME is composed of immune cells, endothelial cells, fibroblasts and various signaling molecules such as chemokines [8]. The push and pull between pro- and anti-tumorigenic signaling pathways create a challenging environment to study, treat and fully understand. Add to this the individual characteristics of different cancer phenotypes and the goal of identifying overarching, unifying concepts that will apply to many cancers becomes even more difficult. Hypoxia has been shown to be universally associated with many tumor types as the natural metabolic profile of an evolving tumor is characterized by critical oxygen depletion, extracellular acidosis, elevated levels of adenosine and lactate and deprivation of essential nutrients [1]. Additionally, hypoxia contributes to intratumoral heterogeneity, metastatic progression, genetic instability, angiogenesis and the evolution of therapy-resistant clones [1,8]. For this reason, it is apparent why so many researchers have sought to target hypoxia pathways to mitigate immunosuppression and improve therapeutic outcomes. 

## 2. Hypoxia Signaling and Metabolism

### 2.1. HIF Signaling Pathways

**Figure 1:**
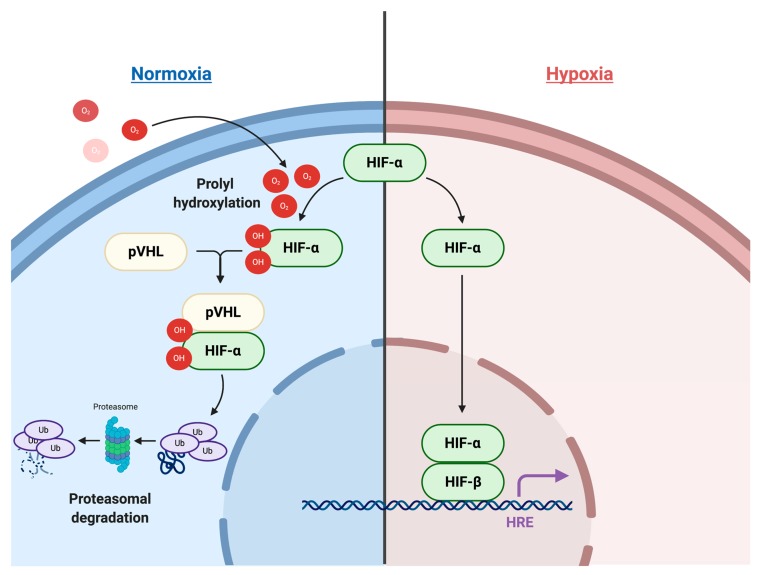
Hypoxia-inducible factor (HIF) signaling pathway.

Hypoxia signaling in the TME involves complex pathways and processes. At the core, the cellular response to hypoxia is mediated by two master regulators that comprise a heterodimeric complex. This complex, formed by a constitutively expressed nuclear HIF-1β and a cytoplasmic oxygen-dependent HIF-α (HIF-1α, HIF-2α and HIF-3α), is further stabilized by a group of oxygen- and iron-dependent enzymes known as HIF-prolyl hydroxylase domain enzymes (PHD1-3) [9]. Under normative conditions, PHDs hydroxylate two prolyl residues of the HIF-α subunit, initiating binding of the Von Hippel-Lindau tumor-suppressor protein (pVHL) and subsequent ubiquitination and proteasomal degradation. However, under hypoxic conditions, PHDs are suppressed and HIF-α subunits translocate into the nucleus to bind with HIF-1β. The heterodimeric HIF-α:HIF-1β transcription factor complex then locates to the hypoxia-responsive elements (HREs) of its target genes, resulting in their transcriptional upregulation (Figure 1).

**Figure 2:**
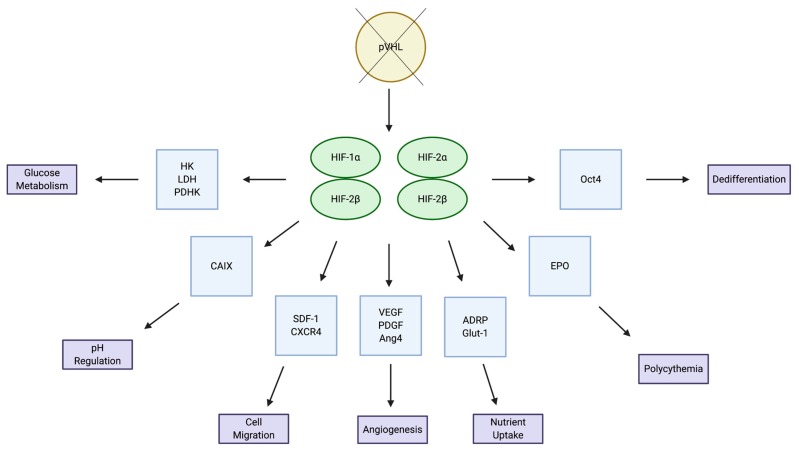
Overlapping and distinct target genes and pathways for HIF-1a and HIF-2a.

There are three HIF-α subunits: HIF-1α, HIF-2α and HIF-3α. Of the three subunits, HIF-1α and HIF-2α are the most well studied and share 48% of their amino acid sequence, with similar protein structures [10]. Interestingly, even with their extreme similarities, these two subunits are non-redundant to one another and have both overlapping and distinct target genes and mechanisms of regulation (Figure 2). In one study, Holmquist-Mengelbier et al. demonstrated that the difference between the two subunits lies not only in the genes in which they transcribe, but also in the conditions under which they are stabilized [11]. They propose a temporal shift in HIF utilization, where HIF-1α appears to be most active during the acute phase of hypoxic adaptation and HIF-2α dominates during later, chronic phases of hypoxia. Further to the genetic differences at the transcriptional level between the two subunits, HIF-2α has been identified as the endothelial Per-Arnt-SIM (EPAS1) domain, an endothelium-specific HIF-α isoform suggested to have a more specialized function. Peng and colleagues have shown that EPAS1 plays an important role in controlling vascular remodeling and that the protein level of EPAS1 is regulated by oxygen tension, where hypoxia induces stabilization of the protein [12]. Additionally, Bangoura et al. demonstrated that EPAS1 overexpression is directly correlated with tumor size, vascular endothelial growth factor (VEGF) expression and initiation of angiogenesis [13]. 

### 2.2. Metabolic Changes in the TME under Hypoxic Conditions 

**Figure 3:**
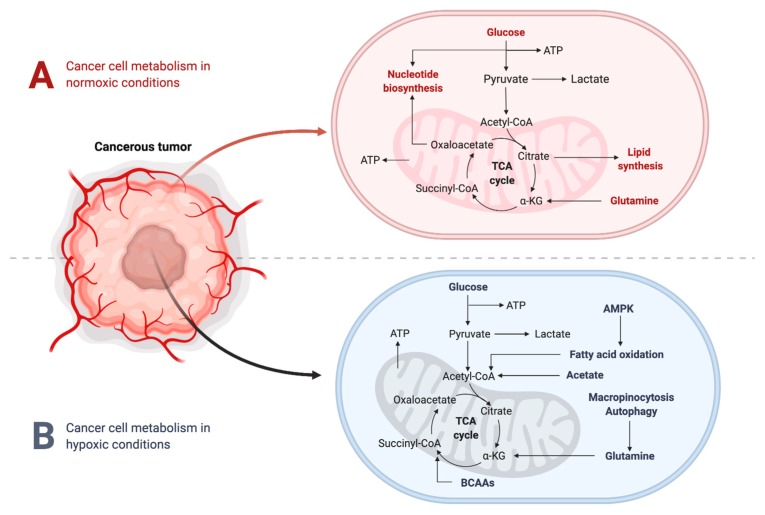
Cancer cell metabolism under hypoxic and normoxic conditions.

It is well established that a hypoxic TME is characterized by increased concentrations of lactic acid, due to the “Warburg effect”—the metabolic shift occurring in highly proliferating cells that convert glucose to lactate, even in the presence of oxygen (aerobic glycolysis). During this process, cancer cells predominantly obtain their energy through the glycolytic pathway, rather than the tricarboxylic acid (TCA) cycle (Figure 3). This phenomenon seems inefficient at first glance, due to the fact that glycolysis only produces 2 ATP per molecule of glucose, whereas the TCA cycle is able to produce 36 ATP. However, the glycolytic pathway has significantly faster kinetics, meaning that it can produce a comparable amount of ATP during the same amount of time [14]. This metabolic reprogramming conserves nutrients for synthesis of nucleic acids, lipids and amino acids to support cellular growth, rather than being oxidized in the mitochondria for maximal output of ATP [15,16,17,18]. Further to this, a high rate of glycolysis leads to increased lactate production, which ultimately results in TME acidosis, altering the tumor stroma and increasing invasive potential. 

Studies have shown that oxygen can act as a direct regulator of PHD activity and that CO_2_ production during mitochondrial respiration through the TCA cycle can also suppress HIF activity [19]. While the exact mechanism for HIF suppression is not well elucidated, many studies suggest that acidification inhibits synthesis of the mammalian target of rapamycin (mTOR), a protein kinase that is a core regulator of cellular processes [20]. Indeed, hypoxia inhibits downstream signaling and mRNA translation initiation of mTOR, resulting in tumor progression and hypoxia tolerance in advanced tumor settings. Prolonged hypoxic exposure can also lead to endoplasmic reticulum (ER) stress, which activates the unfolded protein responses (UPR). Similar to mTOR signaling, this hypoxia-induced cascade results in several downstream effector pathways that function together to promote hypoxia tolerance. Ultimately, hypoxia-mediated signaling through mTOR and the UPR can have profound influences on gene expression and cellular behavior [9]. 

## 3. Hypoxia and the Immune System

In hypoxic regions in the TME, cancer cells are able to adapt and support cellular growth and proliferation through the production of metabolic intermediates that can act as precursors for biosynthetic pathways. However, these oxygen-deprived conditions have been shown to reduce activation levels of tumor-infiltrating lymphocytes (TILs), resulting in immunosuppression and evasion of immune detection [3]. Long proposed as a viable pathway to target for immunostimulatory therapies, many researchers have developed therapeutic pathways for blocking hypoxia-associated transcription factors. In particular, upregulation of HIF-1α has been heavily implicated in cancer biology [21] and is shown to affect varying aspects of the anti-tumor immune response, including the differentiation and function of immune cells within the TME [1]. There are many different immune cell populations that are key to mounting an effective anti-tumor immune response. Disruption of any of these cellular populations can shift or diminish the immune response, allowing tumors to evade detection and escape immune-mediated killing. In this section, we will review some key immune populations and how their functions are altered under hypoxic conditions (Table 1). 

### 3.1. Dendritic Cells 

Dendritic cells (DCs) represent a heterogenous population of immune cells that infiltrate the TME to process and present antigens to naïve T cells. While DCs are only one of many antigen-presenting cell types, they are a key link between the innate and acquired immune response, as they are involved in the initiation of both stages of immunity. While DC maturation is unaffected in hypoxic conditions, their differentiation and function are heavily altered. Giovarelli and colleagues have shown that hypoxia inhibits antigen uptake by DCs, changes the DC chemokine expression profile and has profound effects on DC differentiation, adaptation and activation in inflamed tissues such as tumors [22]. This is corroborated by another report demonstrating hypoxia-induced downregulation of a variety of DC differentiation and activation markers, including CD40, CD80 and MHC class II [23]. DCs are also key components of the immunostimulatory cascade and an integral part of generating an immunogenic cell death (ICD)-mediated anti-tumor immune response [24]. For this reason, environmental factors influencing DC function may also change the natural “immunogenicity” of a tumor and, in turn, shift the mechanism of cell killing to be tolerogenic in nature. 

### 3.2. Macrophages 

Macrophages represent key regulators of the complex relationship between the immune system and cancer. In particular, increased levels of tumor-associated macrophages (TAMs) has been implicated in immunosuppression, neovascularization, metastasis and poor responses to cancer therapies [25]. In contrast to these pro-tumorigenic functions, macrophages may also be essential mediators in immune defense, contributing to an effective anti-tumor immune response. These polarizing functions of a singular cell type can be attributed to the fact that macrophages have high levels of plasticity and, as such, their differentiation relies heavily on the microenvironment in which they are found [26]. There are two main types of tissue macrophages: classical M1 and the alternative M2 phenotype. In general, macrophage responses are shaped by the type, each with their own unique actions. Macrophages that are able to function as effector molecules for pathogen recognition and killing are generally M1-like, characterized by the generation of reactive oxygen species (ROS) and nitric oxide (NO), as well as the expression of high amounts of IL-2 and low levels of IL-10 [27]. Alternatively, cytokines such as IL-4, IL-13 and IL-10 can induce macrophages to effectively execute anti-inflammatory, pro-tumorigenic and pro-angiogenic (M2-like) features [27]. Upon assessment of TAMs found specifically in hypoxic niches within the tumor, researchers have found an aggressive M2 phenotype capable of mediating resistance to many anticancer therapies [28]. Similarly, clinical studies of the histological localization of TAMs have demonstrated a clear correlation between TAM infiltration in hypoxic/necrotic tumor niches and worse prognostic outcomes [29].

### 3.3. B Cells 

The humoral immune response relies on the production of antibodies by B lymphocytes and their progeny, plasma cells. While B cells are primarily known for their crucial role in antibody production, they also stimulate the release of a variety of cytokines and contribute to immunomodulatory responses [30,31]. Defects in the B cell development process can lead to immunodeficiency, autoimmunity or malignancy. While not fully understood or well characterized, the significance of hypoxia-mediated channels, and specifically HIF-1α, to B cell developmental and signaling pathways is well established in the literature [32,33]. Studies have shown that HIF-1α is required for hypoxia-induced cell cycle arrest and the absence of HIF-1α in lymphoid tissues of chimeric mice causes disruption of B cell development [34]. HIF-1α also plays a key role in controlling B cell protective activity in autoimmune diseases and in driving IL-10 production [35]. 

Germinal centres (GCs) are well established and studied as the main sites where antigen-activated B cells expand and undergo hypermutation and selection [36]. This process plays a key role in the presentation of antigens on follicular DCs and associated responses to immunotherapy treatments. A recent study published by Jellusova et al. demonstrated that GC B cells increase glycolysis through HIF during metabolic adaptation to hypoxic conditions [37]. They further identified a metabolic sensor, glycogen synthase kinase 3 (Gsk3), as being required for the generation and maintenance of GC B cells, which require high glycolytic activity to support growth and proliferation in a hypoxic TME [37]. Caro-Maldonado and colleagues have shown that B cell-specific deletion of the glucose transporter *Glut1* resulted in decreased B cell proliferation and impaired antibody production [38], further highlighting the need for increased glycolysis to maintain B cell activity in hypoxic microenvironments. In the context of malignant conditions, tumor-associated B cells have been identified as key drivers of the sustained inflammation necessary for therapeutic efficacy [39]. This emphasizes the importance for HIF-1α-B cell interactions in the TME. Overall, as the significance of B cells in mediating responses to immunotherapies gains increasing prominence in the literature, so too does the need to better understand the relationship between hypoxia factors and B cell developmental processes and functions. 

### 3.4. T Cells 

T cells are a type of lymphocyte that develop in the thymus gland and play a central role in the adaptive immune response. During the maturation process, T cells differentiate into CD4^+^ helper T cells and CD8^+^ cytotoxic T cells. Stimulation of CD4^+^ T cells in the TME causes further cell differentiation into the different subpopulations Th1, Th2, Th17 or Treg (regulatory T cells) [40]. The function of T cells in humoral immunity includes critical interactions between B cells and activated extrafollicular CD4^+^ T cells. Well established as a mainstay of GC architecture, hypoxia drives response mechanisms and T cell function in response to the generation of antibodies. In particular, depletion of HIF-1α from CD4^+^ T cells has been shown to reduce frequencies of antigen-specific GC B cells, follicular T helper (Tfh) cells and antigen-specific antibodies [41]. 

In a resting state, naïve T cells require low amounts of glucose, amino acids and fatty acids to sustain basic energy requirements. However, activated T cells require markedly increased energy to fuel the synthesis of macromolecules, intracellular mediators and effector cytokines. This increased energy consumption requires metabolic reprogramming in which active T cells increase glucose and glutamine catabolism for nucleotide and lipid synthesis, while oxidative phosphorylation is maintained for production of ATP [42]. Additionally, T cell receptor (TCR)-CD28 co-stimulation triggers the shift from naïve to effector T (Teff) cells partially through the mTOR pathway and activation of HIF-1α. This promotes glycolytic gene expression and post-translational modification that is an essential driver of aerobic glycolysis and amino acid metabolism in Teff cells [43]. Glycolic inadequacies during metabolic reprogramming can result in T cell anergy or the shunting of potential Teff cells to the Treg lineage [44]. 

Hypoxic TMEs and HIF-1α can directly affect the frequency of CD8^+^ T cells in the TME, leading to immunosuppression due to a lack of cytotoxic cells. Additionally, high lactate levels in the TME have been shown to suppress the mTOR pathway, inhibiting glycolysis and resulting in impaired T cell function [45]. Glycolysis inhibition is also associated with an increased expression of the inhibitory receptor programmed death-1 (PD-1), which is correlated with T cell exhaustion and non-responsiveness, aiding in tumor immune escape [46]. Interestingly, hypoxia can also contribute to an immunostimulatory function, as T cells that survive in hypoxic niches have actually been shown to display increased cytolytic activity [47]. HIF-1α has been shown to play a role in memory CD8^+^ T cells, which persist beyond the initial immune response, outlasting their terminally differentiated effector counterparts. Similar to naïve T cells, memory CD8^+^ cells are quiescent in nature. They can, however, traffic to a diverse range of tissues and mount a rapid response against future antigenic re-challenge. This increase in functional kinetics is characterized by an immediate metabolic transition towards a reliance on aerobic glycolysis, dependent on the phosphatidylinositol-3-kinase/protein kinase B (PI3K/Akt) signaling pathway. Interestingly, Sukumar and colleagues have shown that while inhibition of glycolysis (and, in turn, inhibition of HIF-1α expression) led to shortened effector function, it concomitantly enhanced the generation of memory cells and anti-tumor functionality [48]. 

### 3.5. Natural Killer (NK) Cells 

NK cells are a class of cytotoxic innate lymphoid cells with potent anti-tumor activity. They possess a broad array of receptors that can recognize ligands induced by tumor formation, cellular stress and DNA damage [49]. Through these receptor recognitions, NK cells are able to direct their lytic machinery to target and eliminate malignant cells in the body. NK cells also release many pro-inflammatory cytokines and chemokines that can aid in amplifying an anti-tumor immune response [49]. In hypoxic niches, NK cells undergo significant metabolic reprogramming that alters their phenotypic and functional responses. Balsamo et al. have shown that hypoxia can downregulate expression and function of most NK cell receptors that are directly responsible for exerting cytolytic activity against tumor cells [50]. In an interesting study, Krzywinska and colleagues demonstrated that HIF-1α depletion impairs NK cell function and tumor growth. This finding was in direct correlation with decreased levels of VEGF, identifying NK cells as an inhibitor of angiogenesis in response to hypoxic conditions [51]. Further data has illustrated that activation of the PI3K/mTOR signaling pathway is critical for HIF-1α upregulation in NK cells, providing a molecular basis for reduced NK cell functions [52]. 

### 3.6. Myeloid-Derived Suppressor Cells (MDSCs) 

One of the most well-studied yet poorly understood immune populations is that of MDSCs. MDSCs are bone marrow-derived myeloid progenitors and one of the largest contributors to immunosuppression in the TME. As well as directly suppressing T cells, NK cells and DCs, they also aid in evasion of immune detection. MDSCs normally differentiate into granulocytes, macrophages or DCs [53], but in abnormal pathological conditions such as cancer they have been shown to maintain their undifferentiated state and rapidly undergo expansion [54]. Corzo et al. detailed the role of hypoxia pathways in MDSCs and showed that HIF-1α is responsible for MDSC differentiation and function in the TME [55]. Additionally, hypoxia can enhance MDSC migration to the tumor site directly via HIF-1α-mediated chemokine production [56]. Chiu and colleagues eloquently demonstrated hypoxia as a central driver of MDSC accumulation in tumors through their secretion of various chemokines such as CCL26 [57]. In a follow-up study, Chiu and colleagues further showed that knockdown of CCL26 profoundly reduces MDSC recruitment, angiogenesis and tumor growth [58]. Hypoxia also aids in MDSC-driven metastasis by influencing the seeding of MDSCs in the pre-metastatic niche through secretion of lysyl oxidase [59]. This process is a key component in the development of metastatic lesions. This highlights the detrimental effects hypoxia can have in promoting these highly immunosuppressive, pro-tumorigenic cell types. 

## 4. Hypoxia and Immunogenic Cell Death

Since the initial descriptions of cell death dating back to the mid-19th century [60], researchers have abandoned the singular postulation that cell death is a uniform, regulated process that serves only in the maintenance of homeostatic conditions and goes undetected by the immune system. Instead, as new, unique forms of cell death have been identified and described in the literature, researchers have begun to delve deeper into the associated biology of each type and, in particular, their quantifiable functional features [61,62,63]. A good example of this paradigm shift in classification is the streamlined and well-studied process of apoptosis. Though apoptosis has historically been thought to be an “immune-quiet” form of cell death, more investigation in recent years has shown that apoptotic cells can in fact be detected by the immune system and elicit an antigen-specific adaptive immune response [64,65,66,67].

ICD is a form of cell death characterized by the chronic release of damage-associated molecular patterns (DAMPs) in the TME [64,68]. Hallmarks of classic ICD include the release of immunomodulatory molecules such as high mobility group box 1 (HMGB1) and adenosine triphosphate (ATP) and surface expression of calreticulin (CR). The quantity of these DAMPs in the TME correlates closely with the “immunogenicity” of a tumor and, in turn, its ability to kill cells through bona fide ICD. Currently, the gold-standard strategy used to evaluate the ability of a specific stimulus to cause true ICD relies on vaccination assays [60,64]. In brief, cancer cells are exposed in vitro to the stimulus and dying cells are inoculated subcutaneously into the flank of immunocompetent mice, prior to tumor implantation with live cells of the same type. Mice are monitored for tumor growth and the number of mice that do not develop tumors is a direct reflection of the degree of immunogenicity of cell death induced by the chosen stimulus [60].

CR, a resident chaperon protein predominantly located in the endoplasmic reticulum (ER), is of particular interest when it comes to hypoxia. Han and colleagues recently showed that hypoxia induces cell surface exposure of CR in human and murine breast cancer cell lines, in an ER stress-dependent manner [7]. In line with these findings, Olin and colleagues hypothesized that reducing oxygen tension when culturing tumor cells would increase the efficacy of tumor cell lysate vaccines [69]. Indeed, upon vaccinating mice bearing orthotopic glioma and breast carcinoma with lysates cultured in 5% O_2_ (as opposed to the regular level of 20%), mice survived significantly longer, displayed enhanced antigen-specific T cell activation and increased cross-presentation of exogenous antigens. To our knowledge, this is the first paper to eloquently demonstrate the use of tissue culture oxygen levels as an “immunologic switch” to dictate the cellular and humoral immune responses elicited by tumor cell lysates [69]. As a follow up to their initial studies, Olin et al. further investigated the effects of physiological oxygen levels in the development of tumor vaccines, this time assessing DCs as a viable vaccination platform. They showed that gene expression patterns in primary glioma cultures established at 5% O_2_ more closely resembled patient tumors in situ and known immunogenic antigens were more highly expressed. Furthermore, DCs treated with tumor lysates generated from primary tumor cells cultured in 5% O_2_ showed improved antigen-presentation capabilities and increased CD8^+^ T cell tumoricidal activity [70]. In these novel reports, Olin and colleagues have inadvertently demonstrated that physiological oxygen levels induce ICD, as shown through the gold-standard vaccination assay.

Photodynamic therapy (PDT) has also been demonstrated to kill cancer cells through the manipulation of oxygen levels to generate ROS, which induces ER stress-mediated anti-tumor immunity and even the killing of distant metastatic lesions [71,72,73]. Conversely, many studies have shown low levels of oxygen in the TME to be a deterrent to efficacious outcomes with PDT [74,75]. In an effort to switch the effects of a hypoxic TME on PDT, Chen and colleagues developed a hybrid protein oxygen nanocarrier with chlorine e6 (a photosensitizer with anti-tumor activity) encapsulated (C@HPOC) for oxygen self-sufficient PDT. C@HPOC relieved tumor hypoxia, showed increased efficiency over PDT alone and increased infiltration of tumor-infiltrating CD8^+^ T cells. Additionally, in 4T1 murine breast tumor cells, C@HPOC-mediated PDT successfully enhanced ICD through the increased exposure of CR as well as secretion of HMGB1 and ATP [76].

Hypoxia can interfere with a variety of homeostatic regulators within cells, including the UPR and autophagy. Autophagy is of particular interest, as it contributes to the expression of ICD-associated damage-associated molecular patterns (DAMPs), such as CR [77,78]. The role of hypoxia in regulating the UPR and autophagy is quite controversial, with several reports indicating that hypoxia can inhibit or induce autophagy. Indeed, some reports find that hypoxia can induce autophagy in an mTOR-independent manner [79,80]. This supports the notion that hypoxia may potentiate ICD through autophagy-mediated release of DAMPs, DC maturation and the subsequent release of tumor-associated antigens [77]. On the other hand, some reports show that hypoxia prevents autophagy through inhibition of the mTOR pathway [81,82]. One study performed by Li et al. demonstrates enhanced CR expression upon inhibition of late-stage autophagy [83]. It will be important for future studies to elucidate the effects of hypoxia on autophagy within the TME and identify potential immunostimulatory effects that can be exploited for use as immunotherapies.

While hypoxia is generally associated with worse prognostic outcomes, selective studies have also shown that oxygen-deprived TMEs create the inflammatory setting conducive to cells dying via ICD. This finding, while not extensively studied in the literature, highlights the complexity of hypoxia-mediated changes in the TME and the importance of addressing the opposing pro- and anti-tumorigenic nature of hypoxia-driven pathways.

## 5. Hypoxia-Mediated Therapeutic Resistance

As seen with chemotherapy, resistance to immunotherapy can arise in many forms of cancer. Primary resistance can be seen in patients who do not respond to treatment, indicating the inability to generate a robust anti-tumor immune response. However, even when patients respond to therapy, acquired resistance can occur, in which patients relapse after a period of tumor regression. Both primary and acquired resistance can occur as a result of tumor cell intrinsic and extrinsic factors, as described by Sharma et al. [84]. Examples of tumor cell intrinsic factors include the ability of some cancer cells to alter pathways involved in antigen presentation, thereby preventing the initial priming of an anti-tumor response. The most straightforward example of tumor cell extrinsic factors comes in the form of immunosuppressive cells within the TME, including Tregs and MDSCs, which were discussed previously. In this section, we will discuss the role of hypoxia in mediating both primary and acquired forms of resistance to cancer immunotherapy, which can be either intrinsic or extrinsic to the cancer cells.

### 5.1. Hypoxia-Mediated Primary Resistance

As described above, one of the most prominent mechanisms of primary resistance to immunotherapy is the alteration of antigen presentation pathways. Indeed, downregulation of MHC class I (MHC-I) expression and other antigen presentation machinery is a common strategy developed by malignant cells to avoid detection by the immune system [85,86,87]. Several reports have demonstrated how hypoxia can mediate the downregulation of MHC-I in malignant cells [88,89,90]. In one such report, Marjit and colleagues show that the combination of hypoxia and glucose deprivation prevents interferon gamma (IFNγ)-mediated upregulation of MHC-I in B16F10 and TC1 murine cancer cells [91]. This is a result of disrupted IFNγ-mediated phosphorylation of signal transducer and activator of transcription 1 (STAT1), thereby preventing the transcription of its target molecules, including MHC-I and transporter associated with antigen processing 1 (TAP1). Furthermore, PI3K was highly activated under hypoxic/glucose-deprived conditions, and inhibition of PI3K using small molecule inhibitors restored antigen presentation and CD8^+^ T cell recognition of both B16F10 and TC1 cell lines [91]. Similar studies have demonstrated how hypoxia causes the downregulation of MHC-I, TAP1/2 and LMP7 in human renal carcinoma cells in a HIF-dependent manner [88]. Another well-known mechanism of primary resistance to immunotherapy is the upregulation of immune checkpoint molecules on tumor cells. One such immunosuppressive molecule, programmed death-ligand 1 (PD-L1), binds to the inhibitory programmed death 1 (PD-1) receptor on T cells and inhibits their activation and cytotoxic functions [92]. Interestingly, *PD-L1* is now considered to be a target gene of HIF-1α and HIF-2α, indicating a crucial role of intratumoral hypoxia in the regulation of this immunosuppressive ligand [93,94,95,96]. One study demonstrated that PD-L1 levels decreased in 786-O human renal carcinoma cells upon HIF-2α siRNA knockdown. Furthermore, *PD-L1* expression was highly upregulated in cells with HIF-2α overexpression [97]. This observation, however, is not limited to cancer cells within the TME. A study spearheaded by Noman et al. found that hypoxia significantly increases *PD-L1* expression on MDSCs, macrophages, DCs and tumor cells in the TME [94]. This upregulation was shown to be dependent on HIF-1α, but not HIF-2α [94]. The discrepancies between studies indicate that the roles of HIF-1α and HIF-2α in mediating *PD-L1* expression may depend on the cell type and type/location of the tumor. Future research should focus on delineating the role of these transcription factors in regulating PD-L1 expression in different tumor models. Another immune checkpoint molecule, cytotoxic T-lymphocyte-associated protein 4 (CTLA-4), is expressed on activated T cells and inhibits T cell activation when bound to its ligand, CD86, on antigen-presenting cells (APCs) such as DCs [98,99]. Similar to PD-L1, hypoxia has been shown to upregulate the expression of CD86 on DCs in a HIF-1α-dependent manner [100]. As mentioned previously, it is well known that hypoxia can shift the metabolic state within a tumor. Several reports have shown that cancer cells can adapt their metabolism to thrive under hypoxic conditions, allowing cancer cells to metabolically outcompete tumor-infiltrating T cells for glucose, resulting in the inhibition of T cell activity and increased cancer progression [101,102]. Indeed, the metabolic stress in the TME can negatively impact the immune functions of several immune cells in the tumor, including T cells, macrophages and MDSCs [9]. These studies imply that the HIF transcription factors may be suitable therapeutic targets for preventing immunosuppression in the TME, a concept that will be further discussed later in this review.

### 5.2. Hypoxia-Mediated Acquired Resistance

Resistance to immunotherapy can still arise in patients who briefly respond to therapy. This form of acquired resistance can often be attributed to tumor heterogeneity. In the case of immunotherapy, response against specific antigens exerts selective pressure towards antigen-loss cancer cells over time, a concept termed antigen escape [103]. This phenomenon has been observed in patients with triple-negative breast cancer (TNBC) during neoadjuvant chemotherapy (NAC), in which TNBC persisted after treatment in half of the patients. Upon further analysis using single-cell DNA and RNA sequencing, the data indicated that the resistant clones were pre-existing and adaptively selected by NAC [104]. Similar findings were seen in patients with stage IV melanoma treated with adoptive T-cell transfer [105], and even in murine models harboring B16 melanoma tumors [106]. As with other forms of resistance, there is accumulating evidence that hypoxia can potentiate intratumoral heterogeneity. The expression of genes involved in mismatch repair and homologous recombination are downregulated under hypoxic conditions [107,108,109]. This, in turn, can drive genomic instability and mutagenesis which can increase the probability of creating resistant clones [110,111,112]. Aside from generating resistant clones in the primary tumor, genomic instability can also result in the formation of metastasis. HIF-1α is heavily associated with metastasis formation and has been shown to drive several steps of metastasis, including epithelial-mesenchymal transition (EMT), invasion and extravasation [113]. One report investigating the invasion and metastasis of esophageal carcinoma Eca109 cells shows that HIF-1α inhibits the tumor suppressor E-cadherin and upregulates the expression of matrix metalloproteinase-2 (MMP-2), a protein involved in enabling the migration of cells from the primary tumor to sites of metastasis [114]. Another, similar study performed by Zhao et al. finds that the actin-binding protein LIM and SH3 domain protein 1 (LASP1) is upregulated by HIF-1α and is critical for metastasis formation of several human pancreatic cancer cell lines [114]. Angiogenesis is also essential for the dissemination and establishment of tumor metastases, since the ability of metastatic clusters to access blood vessels allows for their migration [115,116]. One of the key roles of HIF-1α includes its regulation of angiogenic factors such as vascular endothelial growth factor (VEGF). HIF-1α has been shown to drive VEGF-mediated angiogenesis within the TME in a variety of cancers, including ovarian, pancreatic and breast cancers [117,118,119,120]. Furthermore, HIF-1α-mediated proangiogenic signaling is not limited to cancer cells, and can occur in other cell types within the TME, including cancer-associated fibroblasts (CAFs) [121].

**Figure 4:**
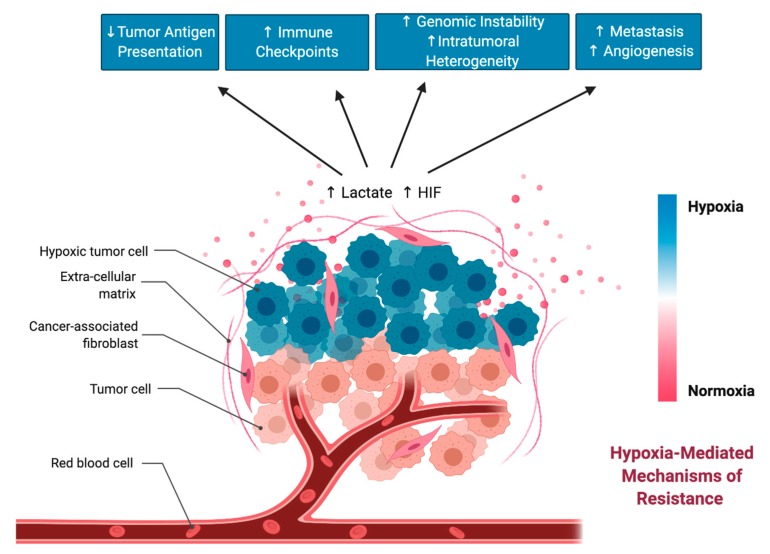
Hypoxia-mediated changes in the tumor microenvironment (TME) that can drive resistance to immunotherapy.

Based on the studies highlighted in this section, hypoxia and HIF transcription factors heavily contribute to different forms of resistance to immunotherapy (Figure 4). This is in line with studies that correlate high HIF expression with poor prognostic outcomes in several cancers [122,123,124]. Altogether, selective targeting of HIF in tumors is an attractive therapeutic approach that may bolster immunotherapeutic agents and prevent hypoxia-mediated resistance to immunotherapy.

## 6. Hypoxia-Targeted Immunotherapies

### 6.1. Strategies for Targeting Hypoxia-Induced Pathways

The concept of targeting hypoxia-induced pathways as a cancer therapy is well established [125,126,127,128,129]. Indeed, several candidates for HIF inhibitors are currently being tested in phase I and phase II clinical trials as cancer therapeutics [130]. Most hypoxia-targeting therapies, however, are focused on disrupting metabolism and angiogenesis in the tumor to suppress tumor progression and the formation of metastasis [131,132]. Unfortunately, HIF inhibitors have poor selectivity, and so therapies often involve inhibition of downstream pathways or the use of hypoxia-activated prodrugs. In this section of the review, we will discuss the potential of targeting hypoxia-mediated pathways to potentiate cancer immunotherapy. As discussed previously, hypoxia has been shown to mediate many forms of resistance to immunotherapy. This provides good rationale for combinatorial approaches for immunotherapy and inhibition of HIF pathways.

Hypoxia can play an important role in the regulation of immunosuppressive molecules and the activation of immunosuppressive cells such as Tregs and MDSCs. Therefore, reducing hypoxia in the tumor may prevent suppression of an anti-tumor immune response. Indeed, a few promising pre-clinical studies support the potential for combinatorial therapies involving immunotherapy and hypoxia-based therapies. One such study includes the use of the hypoxia-activated prodrug, TH-302. This study demonstrates that TH-302 reduces hypoxia in a murine prostate tumor model, and can cure up to 80% of tumor-bearing mice when combined with immune checkpoint inhibitors [130]. Furthermore, the combination reduced MDSC density in the tumor by 50% [130]. Another example published in Nature Communications describes the HIF-1-mediated expression of ectoenzyme, ectonucleoside triphosphate diphosphohydrolase 2 (ENTPD2), which promotes the maintenance of MDSCs in a murine hepatocellular carcinoma model [130]. The combination of ENTPD2 inhibitors and immune checkpoint inhibitors significantly increased the infiltration of T cells into the tumor and prolonged survival of tumor-bearing mice, when compared to using immune checkpoint inhibitors alone [130]. Another group reduced intratumoral hypoxia using the type II diabetes drug, Metformin, and found that combination with PD-1 blockade improved anti-tumor T cell function and tumor clearance in B16 and MC38 murine tumor models [130].

Inhibition of HIF transcription factors can also be achieved by targeting the PI3K/AKT/mTOR pathways [133,134,135]. Molecular regulators of mTOR, such as Tuberous sclerosis complex 2 (TSC2) have been implicated in HIF-1α. TSC2 knockouts resulted in increased HIF-1α accumulation and the upregulation of HIF-induced genes such as *VEGF*. Interestingly, the mTOR inhibitor, rapamycin, reduced HIF-1α levels in TSC2 knockouts, although only partially reducing VEGF levels [134]. Other studies have found that the PI3K/AKT/mTOR pathways play a role in the regulation of PD-L1 in several murine cancer models, including non-small-cell lung cancer [136], colorectal cancer [137], pancreatic cancer [138] and breast cancer [139]. Furthermore, both rapamycin and the AKT inhibitor, MK-2206, inhibit the expression of *PD-L1* in breast cancer cells [139], although it is unclear if this regulation occurs in a HIF-dependent manner. Other strategies include targeting factors downstream of HIF-1α, such as VEGF, to reduce immunosuppression and sensitize cells to immunotherapy [140]. One study in PNAS demonstrated that low doses of anti-VEGF2 antibody polarizes TAMs from their immunosuppressive M2 phenotype into an immunostimulatory M1 phenotype. This results in improved tumor infiltration of CD8^+^ and CD4^+^ T cells [141].

### 6.2. Considerations for Targeting Hypoxia-Induced Pathways

While targeting hypoxia-induced pathways in combination with immunotherapies is an attractive approach, it should be carefully considered, as some immunotherapies may benefit from the hypoxic conditions within the TME. Oncolytic viruses are gaining traction as a promising form of immunotherapy, with an FDA-approved oncolytic Herpes Simplex Virus-1 (HSV-1) already available as a first-line treatment for melanoma in North America. One study demonstrates that oncolytic HSV-1 replicates more efficiently in hypoxic tumors, and that oxygenation of subcutaneous tumors in mice results in reduced replication in the tumor [142]. This study is corroborated by a similar finding in which hypoxic conditions promoted the replication of oncolytic HSV-1 in otherwise resistant breast cancer cells [143]. Interestingly, the effect of hypoxia on therapeutic efficacy with oncolytic virotherapy depends on the virus. For example, oncolytic adenovirus replication is hampered under hypoxic conditions, while oncolytic HSV-1 replication is enhanced under hypoxic conditions. Oncolytic Vaccinia virus demonstrates improved cytotoxicity in hypoxic cancer cells, but has no increase in transgene expression [144]. Some studies have also demonstrated that inhibition of the mTOR pathway can also be detrimental for immunotherapy. Rapamycin and other mTOR inhibitors may have immunosuppressive effects on a variety of immune cells. This includes reduced activation and antigen presentation by dendritic cells [145,146,147], reduced CD8^+^ T cell infiltration into the tumor [148], and increased Treg expansion [149,150].

Therefore, pre-clinical data provides promising evidence for the potential of targeting hypoxia-induced pathways in combination with immunotherapy. However, such combinations must be carefully considered. It is pertinent that future research focuses on the effects of targeting hypoxia when combined with different forms of immunotherapy. While there is potential for reducing hypoxia-mediated resistance to immunotherapy, some therapies may be hampered in oxygenated tumors, resulting in reduced therapeutic efficacy.

## 7. Conclusions

The TME is a complex network composed of immune cells, endothelial cells, fibroblasts and various signaling molecules. While scientific literature and clinical studies predominantly address the immunosuppressive nature of hypoxia-driven pathways, they often fail to acknowledge the potential of the resultant inflammatory microenvironment to promote cells dying via bona fide ICD. In this review, we have objectively assessed the role of hypoxia in both pro- and anti-tumorigenic pathways and identified ways in which hypoxia-mediated therapeutic resistance may be overcome. Furthermore, we have highlighted current strategies for targeting hypoxia with immunotherapy treatments. Development of future immunotherapy platforms targeting hypoxia signaling pathways should take into consideration not only the immunosuppressive nature of hypoxia, but also the potential to increase ICD through hypoxia-mediated inflammation.

## Figures and Tables

**Table 1:** Changes in immune cell phenotypes and secretome during hypoxia.

Cell Type	Changes in Function and Secretome	References
DCs	↓ Antigen uptake	[22,23]
	↓ CD1a, CD40, CD80, CD83, CD86, MHC class II	
Macrophages	↓ M1 phenotype	[27,28,29]
	↑ M2 phenotype	
	↓ IL-2	
	↑ IL-4, IL-10, IL-13	
B cells	↑ Development	[34,35]
	↑ IL-10	
T cells	↓ Cytotoxic function	[41,44,48]
	↑ Anergy	
	↑ Treg expansion	
	↑ Memory function	
	↑ Antibody production	
	↑ PD-1	
NK cells	↓ Cytolytic activity	[51,52]
	↑ VEGF	
MDSCs	↑ Differentiation and function	[55,56,59]
	↑ Recruitment to tumor site	
	↑ Extracellular remodeling	
